# Phenotyping and a genome-wide association study of elite lines of pearl millet

**DOI:** 10.1270/jsbbs.23082

**Published:** 2024-07-05

**Authors:** Kota Kambara, Shashi Kumar Gupta, Tetsuo Takano, Daisuke Tsugama

**Affiliations:** 1 Asian Research Center for Bioresource and Environmental Sciences (ARC-BRES), Graduate School of Agricultural and Life Sciences, The University of Tokyo, 1-1-1 Midori-cho, Nishitokyo-shi, Tokyo 188-0002, Japan; 2 International Crops Research Institute for the Semi-Arid Tropics (ICRISAT), Hyderabad, Telangana State, India

**Keywords:** pearl millet, *Pennisetum glaucum*, elite lines, GWAS, PAL2

## Abstract

Pearl millet (*Pennisetum glaucum* (L.) R. BR.) is a cereal crop mainly grown in India and sub-Saharan Africa. In pearl millet, genes and genomic regions associated with traits are largely unknown. Pearl millet parental lines bred at the International Crops Research Institute for the Semi-Arid Tropics (ICRISAT) are useful for the production and breeding of pearl millet. However, the phenotypic diversity of these lines has not been fully evaluated. In this study, 16 traits of 107 of those parental lines were assessed with field trials in Japan, and a genome-wide association study (GWAS) was performed using these phenotypic data to identify the genomic regions and genes associated with those traits. The GWAS revealed genomic regions associated with culm height and pigmentation of the shoot basal part (PS). The genomic region associated with PS contained a homolog of *PHENYLALANINE AMMONIA LYASE 2* (*PAL2*), a gene involved in anthocyanin accumulation in *Arabidopsis thaliana*. The *PAL2* homolog can be a candidate for a gene involved in regulating PS in pearl millet. These results provide a better understanding of the phenotypic diversity of pearl millet and its genetic background.

## Introduction

Pearl millet (*Pennisetum glaucum* (L.) R. BR.) is a cereal crop mainly grown in India and sub-Saharan Africa. Pearl millet has a high tolerance to drought, heat, and poor nutrient conditions. Thus, it is an important crop for farmers who do not have access to adequate water resources or agricultural inputs.

For some pearl millet traits such as culm height (CH), plant pigmentation, and panicle length, quantitative trait locus (QTL) analyses have been performed to identify genomic regions involved in regulating these traits. Two of these analyses have shown that the purple pigmentation of leaves is controlled by a single dominant locus named *P* (Appa Rao *et al.* 1988, Azhaguvel *et al.* 2003). A recessive *d2* locus located on chromosome 4 is associated with CH (Azhaguvel *et al.* 2003, Burton and Forston 1966, Burton *et al.* 1969). Fine-mapping has shown that a pearl millet homolog of the sorghum *ABCB1* gene, which encodes a P-glycoprotein facilitating cell-to-cell polar auxin transport and which is a causal gene of the dwarf mutant *dw3* in sorghum, is located in the *d2* locus and is thought to act as a dwarfing gene in pearl millet (Parvathaneni *et al.* 2013, 2019). However, the genomic regions and genes associated with pearl millet traits are largely unknown. Identifying such regions and genes can help generate pearl millet lines with better performance.

A genome-wide association study (GWAS) is a method to identify genomic regions or genes associated with a trait by comparing trait data with whole-genome DNA polymorphism data from many individuals. The release of the reference genome sequence of pearl millet (Varshney *et al.* 2017) enabled a GWAS to be performed with pearl millet. A GWAS of 281 lines of pearl millet identified a putative zinc/iron-regulated transporter-like protein gene, a putative natural resistance-associated macrophage protein gene, and a putative ferritin-like protein gene as candidates for genes regulating Fe and Zn contents of pearl millet seeds (Pujar *et al.* 2020). Another GWAS identified several genes including a putative leucine-rich repeat receptor-like serine/threonine-protein kinase gene as candidates for genes regulating rhizosheath formation (De la Fuente Cantó *et al.* 2022).

Pearl millet parental lines (A/B lines and R lines: A lines are lines with cytoplasmic male sterility for hybrid production, B lines are maintainer lines for their corresponding A lines and has the same karyotypes as them, and R lines are restorer lines) bred at the International Crops Research Institute for the Semi-Arid Tropics (ICRISAT) are considered useful for the production and breeding of pearl millet for both the private and public sectors (Mula *et al.* 2007). Yield-related traits of some of these lines were previously obtained in India (Gupta *et al.* 2011), and most of these lines were genotyped by restriction site-associated DNA sequencing (RAD-seq) (Varshney *et al.* 2017). However, little is known about the phenotypes of these lines. Learning more about the phenotypes of pearl millet will help to better understand and use their genetic diversity.

Here, we report phenotypic data for 16 traits of 107 of those parental lines grown in Japan and the results of a GWAS for identifying genomic regions associated with these traits.

## Materials and Methods

### Plant material

Seeds of the 107 pearl millet parental lines bred at ICRISAT (Supplemental Table 1), which consists of 83 lines as hybrid seed parents (B lines) and 24 lines as restorer parents (R lines) were used in this study.

### Phenotyping

The 107 lines were planted in a randomized complete block design with two replicates with stripe sowing with one meter per line in June 2022 in a field of the Institute for Sustainable Agro-ecosystem of Services at the University of Tokyo, Japan (35.74°N, 139.54°E). They were cultivated with no fertilizer until they senesced. The lines were assessed for 16 traits (Table 1). The measurement methods are described in Supplemental Table 2.

### Correlation analysis and Principal Component Analysis (PCA)

Pearson’s correlation coefficients between different traits were calculated for the phenotypic data obtained in the field. PCA was performed on 12 traits indicated in Table 1. The scripts used in the PCA are available upon request.

### Genotyping and GWAS

RAD-seq data are available for 101 of the 107 lines phenotyped (Varshney *et al.* 2017). These data were downloaded from the NCBI SRA (National Center for Biotechnology Information Sequence Read Archive, https://www.ncbi.nlm.nih.gov/sra) with the accession numbers listed in Supplemental Table 1. The RAD-seq-derived reads were mapped to the reference genome of pearl millet (Varshney *et al.* 2017) with BWA (Li and Durbin 2009), and polymorphisms were detected with Strelka2 (Kim *et al.* 2018). The number of the polymorphisms detected was 32028331 in total. PCA was performed on plink2 (Chang *et al.* 2015) with these polymorphisms as the input to extract 10 PCs (PC1–10) as population structure data. A GWAS was performed with plink2 using the polymorphisms detected in 20 or more lines, the above population structure data and the above phenotypic data (see the “Phenotyping” section) as the input for the linear model y = Gβ_G_ + Xβ_X_ + e, where y is the phenotype vector, G is the genotype/dosage matrix for the current polymorphism, X is the fixed-covariate (i.e., PC1–10) matrix, and e is the error term. Manhattan plots were drawn for the results (i.e., unadjusted *P*-values for the above polymorphisms) of the GWAS with the qqman package on R (https://doi.org/10.1101/005165). For this, only the additive (i.e., non-PC-specific) effect was used for each polymorphism. For the CH and PS phenotypes (Table 1), adjusted *P*-values including false discovery rates based on the Benjamini-Hochberg method (FDRs_BH) (Benjamini and Hochberg 1995) were obtained by the “--adjust” option on plink2, and genes within 1 Mb of the highest peaks in the Manhattan plots were extracted by a Perl script. Annotations for these genes were obtained from TGIF-DB (Tsugama and Takano 2021). The LD block in the position 257014000-258498000 on chromosome 3 was visualized by LDBlockShow (Dong *et al.* 2021). The sequence of the *d2* allele identified by Parvathaneni *et al.* (2019) was downloaded from NCBI GenBank (https://www.ncbi.nlm.nih.gov/nucleotide/) with the accession number MH059799. The position of the *d2* locus in the reference genome was identified with a BLASTN search (Camacho *et al.* 2009). Scripts used are available upon request.

## Results

### Distribution and correlation of the traits

**Fig. 1.**
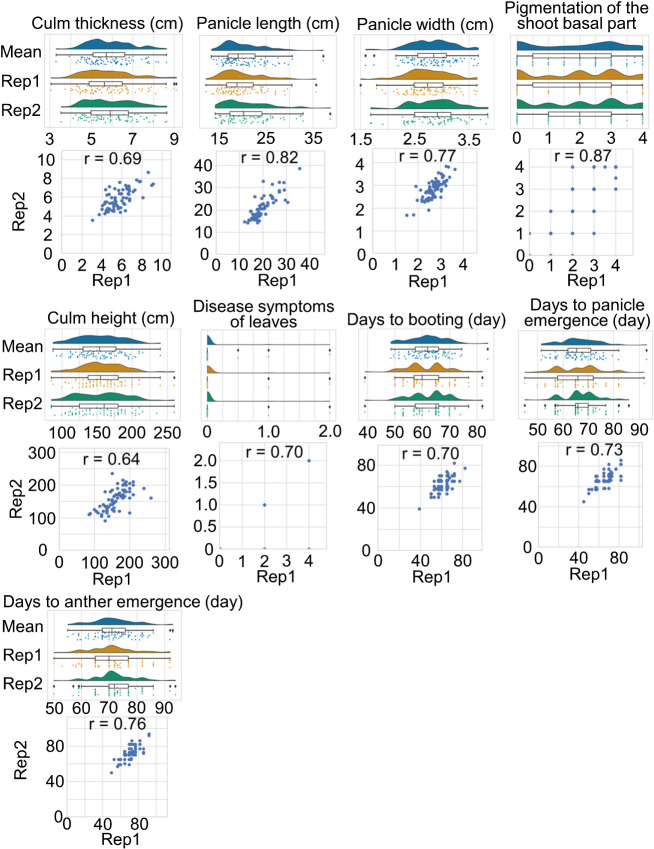
Distribution of phenotypic values and correlation between the two replicates for culm thickness, panicle length, panicle width, pigmentation of the shoot basal part, culm height, disease symptoms of leaves, days to booting, days to panicle emergence, and days to anther emergence. Rep1, replicate 1; Rep2, replicate 2; Mean, the mean value of the two replicates.

Differences were observed in phenotypes for the 16 traits between the 107 pearl millet lines assessed (see Supplemental Fig. 1 for an example of phenotyping and Supplemental Table 3 for all the phenotypic values obtained). Correlation coefficients were high (0.64–0.87) between two replicates for culm thickness (CT), panicle length (PL), panicle width (PW), pigmentation of the shoot basal part (PS), CH, days to booting (DB), days to panicle emergence (DP), days to anther emergence (DA), and disease symptoms of leaves (DS) (Fig. 1). In contrast, correlation coefficients were low (0.13–0.48) between the replicates for plant height at 45 days after sowing (PH45), leaf curliness (LC), and awn growth (AG) (Supplemental Fig. 2). These traits appear to depend on the growth environment. Correlation coefficients were between 0.49 and 0.58 for vigor in the vegetative stage (VI), lodging (LO), and pigmentation of nodes (PN) (Supplemental Fig. 3). CT showed high positive correlation coefficients with PL, PW and DB (0.55, 0.52 and 0.54, respectively) (Supplemental Table 4). This result suggests that late-flowering lines tend to have thicker culms and longer panicles. DB and PH45 showed a negative correlation coefficient (–0.52) (Supplemental Table 4). This result supports the idea that late-flowering lines grow more slowly at the vegetative stage than early-flowering lines.

### Principal component analysis (PCA)

**Fig. 2.**
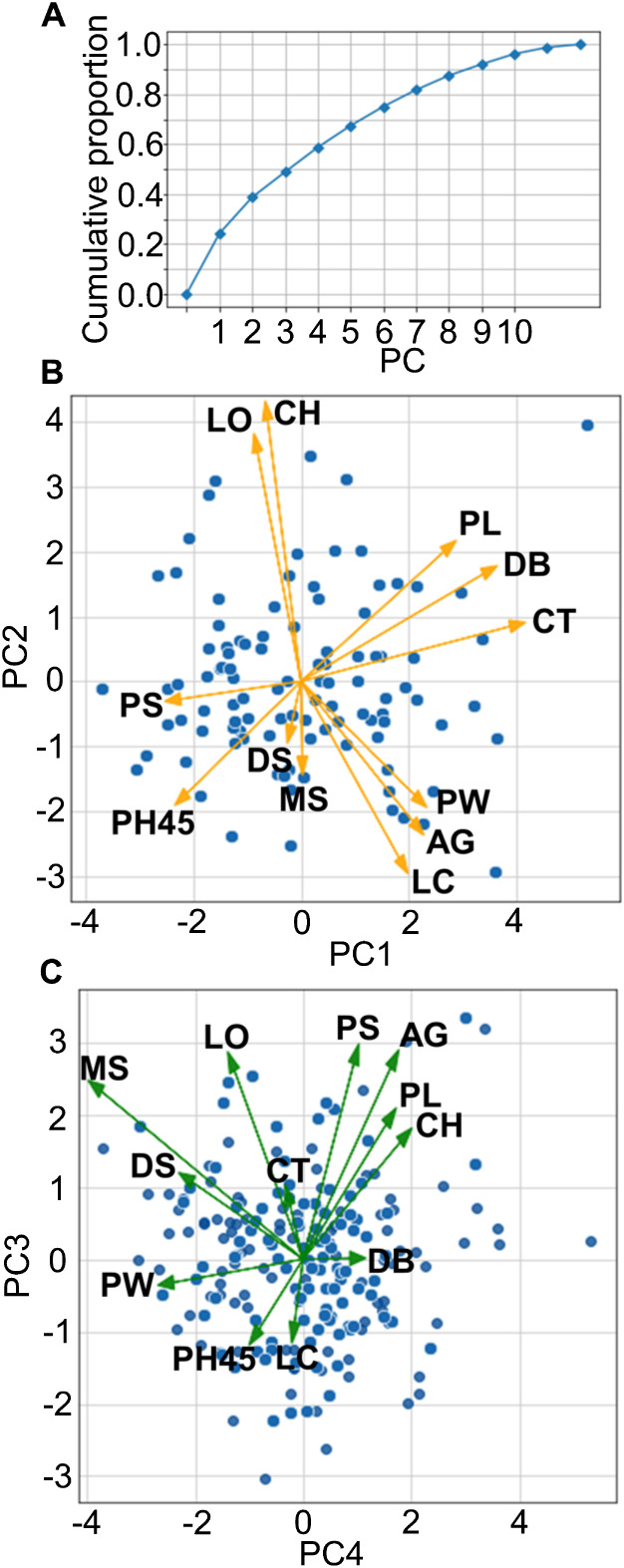
PCA of the 12 traits. (A) The cumulative proportion of each principal component. The x-axis indicates the principal component and the y-axis indicates the cumulative proportion. (B) Principal component scores for the PC1 and PC2. Each blue dot indicates each line and yellow arrows indicate principal component loadings to the PC1 and PC2 for each trait. The x-axis indicates the PC1 and the y-axis indicates the PC2. (C) Principal component scores for the PC3 and PC4. Each blue dot indicates each line and green arrows indicate principal component loadings to the PC3 and PC4 for each trait. The x-axis indicates the PC4 and the y-axis indicates the PC3. PS, Pigmentation of the shoot basal part; LC, Leaf curliness; VI, Vigor in the vegetative stage; DS, Disease symptoms of leaves; CT, Culm thickness; PL, Panicle length; PW, Panicle width; DB, Days to booting; AG, Awn growth; LO, Lodging; PH45, Plant height at 45 days after sowing; CH, Culm height; MS, Milligrams per seed.

PCA was performed with mean phenotypic values of 12 traits, excluding the traits with strong correlations or with some missing values. PC1, PC2, PC3 and PC4 explained approximately 60% of the cumulative proportion (Fig. 2A). Biplots made with the PC1 and PC2 scores (Fig. 2B) and the PC3 and PC4 scores (Fig. 2C) showed no clear clusters. This result indicates that the lines used in this study are phenotypically diverse. In the biplot with PC1 and PC2, the vectors of LO and CH were located close to each other (Fig. 2B), although the correlation coefficient between the LO and CH data was 0.26 (Supplemental Table 4). This suggests that lodging is related to CH in these lines (Fig. 2B).

### GWAS

**Fig. 3.**
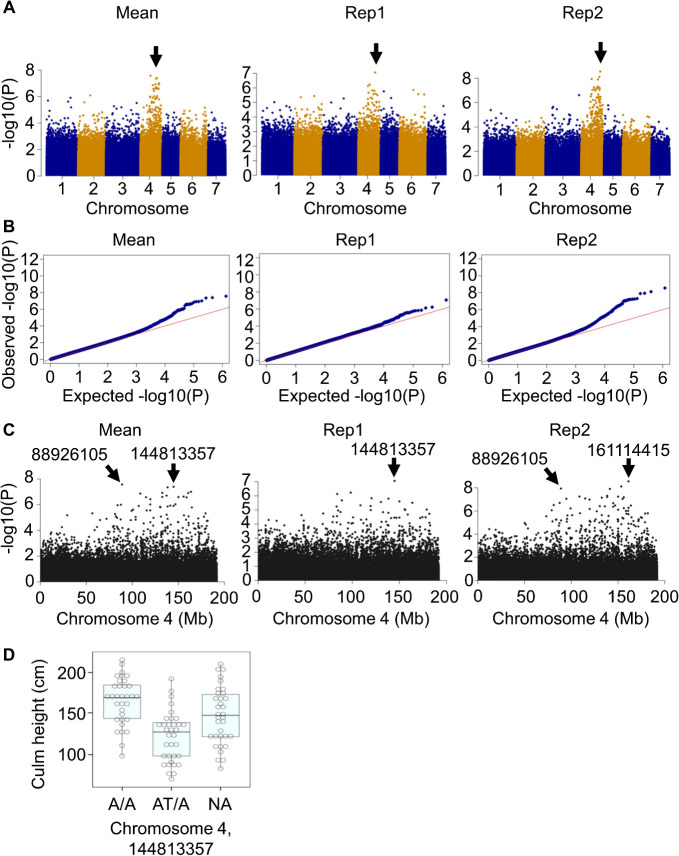
GWAS of culm height. (A) Manhattan plots. Black arrows indicate peaks. (B) Quantile-quantile (QQ) plot. (C) Local Manhattan plot on the chromosome 4. For A–C, Mean, the mean value of the two replicates; Rep1, replicate 1; Rep2, replicate 2. (D) Association of the genotype (or alleles) at the position 144813357 on chromosome 4 with the culm height. The top and bottom edges and the line in the middle of each box indicate the quartiles, and the bar corresponds to the data range. Each dot corresponds to a mean culm height value of a pearl millet line. NA: genotype was not available.

PCA was also performed using the available genotypic data (i.e., 32028331 polymorphisms) to obtain 10 PCs (PC1–10) as indicators of the population structure between the lines used for the phenotyping. In this PCA, ICMB 100117 exhibited an extreme PC1 (Supplemental Fig. 4). Nevertheless, this does not seem to affect GWAS results because the phenotypic values of ICMB 100117 were all average levels (Supplemental Table 3). A GWAS was conducted using the genotypic and phenotypic data and the PC1–10. Polymorphisms strongly associated with CH (with the –log10(P) values higher than 6, for which the FDRs_BH were all lower than 0.03) were detected between the positions 88926105 and 161114415 on chromosome 4 (Fig. 3). However, no homolog of a gene known to regulate CH in any plant species was found in this region. This region was different from the *d2* locus, which corresponds to positions 78949078-79021678 on chromosome 4.

**Fig. 4.**
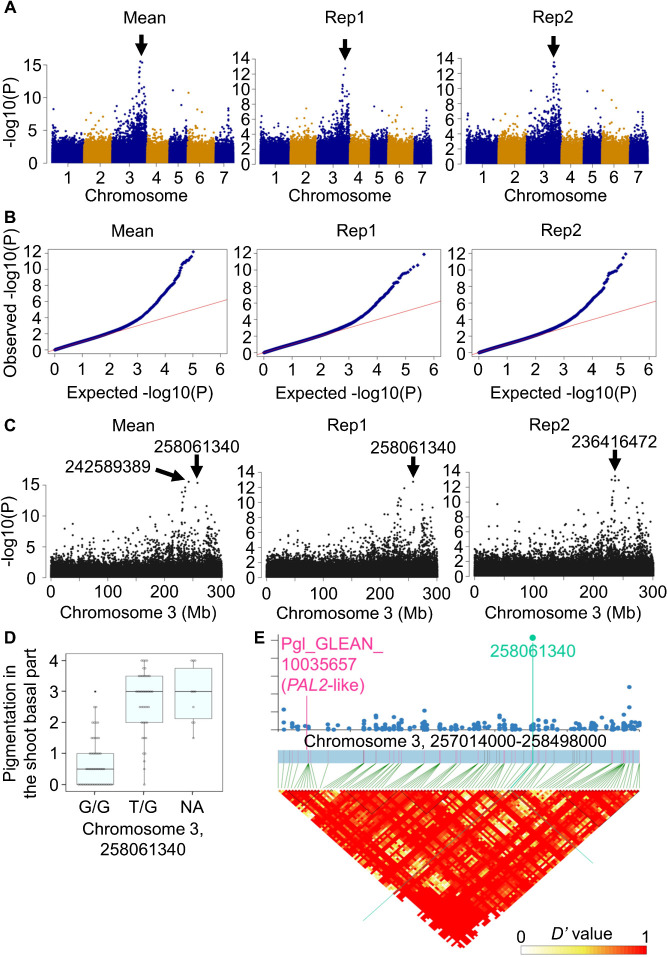
GWAS of the pigmentation of the shoot basal part. (A) Manhattan plots. Black arrows indicate peaks. (B) Quantile-quantile (QQ) plot. (C) Local Manhattan plot on the chromosome 3. For A–C, Mean, the mean value of the two replicates; Rep1, replicate 1; Rep2, replicate 2. (D) Association of the genotype (or alleles) at the position 258061340 on chromosome 3 with the pigmentation of the shoot basal part. The top and bottom edges and the line in the middle of each box indicate the quartiles, and the bar corresponds to the data range. Each dot corresponds to a mean culm height value of a pearl millet line. NA: genotype was not available. (E) Linkage disequilibrium (LD) blocks in the proximity of the position 258061340 and a *PAL2*-like gene, Pgl_GLEAN_10035657 on chromosome 3.

Polymorphisms strongly associated with PS (with the –log10(P) values higher than 11, for which the FDRs_BH were all lower than 0.000001) were detected between the positions 236416472 and 258061340 on chromosome 3 (Fig. 4). This region contains Pgl_GLEAN_10035657, a pearl millet homolog of *PHENYLALANINE AMMONIA LYASE 2* (*PAL2*), which is involved in anthocyanin accumulation in *Arabidopsis thaliana* (Huang *et al.* 2010, Koukol and Conn 1961). The *PAL2* homolog is therefore a candidate for the gene regulating PS in pearl millet. No polymorphism was detected in the 3000-bp promoter and genomic (i.e., exon and intron) region of this gene (position 257131430-257136650 on chromosome 3). However, this can be because the RAD-Seq data were insufficient to detect all the polymorphisms in this region and does not exclude the possibility that this gene regulates the PS in pearl millet. GWASs were performed for the other traits, but no specific genomic region appeared strongly associated with those traits (Kambara and Tsugama 2024, https://doi.org/10.6084/m9.figshare.25100537).

## Discussion

In this study, the 16 traits of the 107 lines bred at ICRISAT were assessed in Japan. Most of these traits were not assessed in other studies of pearl millet. Thus, these new results can help to better understand the phenotypic diversity of pearl millet.

For CH, the detected peak in the Manhattan plot corresponded to a broad region on chromosome 4, and no candidates for CH-regulating genes were identified. Further studies such as complete detection of polymorphisms in this region are necessary to narrow down the number of candidates.

The GWAS in this study identified polymorphisms on chromosome 3 associated with PS (Fig. 4). On the other hand, the *P* locus, which can regulate pearl millet foliage color, is located on chromosome 4 (Azhaguvel *et al.* 2003). A single dominant locus that can control the pigmentation of the internode, leaf sheath, midrib and margin in pearl millet is also located on chromosome 4 (Varalakshmi *et al.* 2012). These findings raise the possibility that the genes regulating the pigmentation are different between tissues in pearl millet. In the proximity of the polymorphisms identified in this GWAS, a *PAL2* homolog is present. PALs are enzymes that catalyze the conversion of phenylalanine to trans-cinnamic acid. Trans-cinnamic acid is converted to polyphenolic compounds such as lignin and flavonoids by downstream reactions (Koukol and Conn 1961). *Arabidopsis* has four *PAL* genes, *PAL1–4* (Raes *et al.* 2003). A double loss-of-function mutant of *PAL1* and *PAL2* exhibits no anthocyanin accumulation in various tissues in *Arabidopsis* (Huang *et al.* 2010). This *PAL2* homolog is therefore a candidate for the gene regulating PS in pearl millet. Although the GWAS in this study failed to a genomic region or polymorphisms strongly associated with PN in pearl millet (Supplemental Fig. 5), the PN showed a high correlation with PS (with the correlation coefficient of 0.45, Supplemental Table 4). Thus, the *PAL2* homolog may also regulate PN in pearl millet. It will be interesting to identify polymorphisms in this gene and to determine whether it regulates PS and PN in pearl millet. Flavonoids including anthocyanins are thought to protect plants from UV and oxidative stress (reviewed by Winkel-Shirley 2002). It will also be interesting to see if the anthocyanin accumulation pattern is associated with the growth of pearl millet.

## Author Contribution Statement

All authors contributed to designing the study. The experimental work and data analysis was performed by KK and DT. Material preparation was performed by SG. The first draft of the manuscript was written by KK. DT revised and reviewed the manuscript. All authors read and approved the final manuscript.

## Supplementary Material

Supplemental Figures

Supplemental Tables

## Figures and Tables

**Table 1.** List of traits analyzed in the field trials

Trait	Code	Units
Awn growth	AG*^a^*	Absent (0) ~ Well grown (5)
Culm height	CH*^a^*	Cm
Culm thickness	CT*^a^*	Cm
Days to anther emergence	DA	Days after sowing
Days to booting	DB*^a^*	Days after sowing
Days to panicle emergence	DP	Days after sowing
Disease symptoms of leaves	DS*^a^*	Absent (0) or Present (1)
Leaf curliness	LC*^a^*	Not curled (1) ~ Well Curled (5)
Lodging	LO*^a^*	Not lodged (0) ~ Lodged (1)
Milligrams per seed	MS*^a^*	Mg
Pigmentation of nodes	PN	Green (0) ~ Purple (5)
Pigmentation of the shoot basal part	PS*^a^*	Green (0) ~ Purple (4)
Panicle length	PL*^a^*	Cm
Panicle width	PW*^a^*	Cm
Plant height at 45 days after sowing	PH45*^a^*	Cm
Vigor in the vegetative stage	VI	Weak (1) ~ Strong (6)

*^a^* These traits were used for the principal component analysis (PCA).
