# Supplementary material for: Phenotyping and a genome-wide association study of elite lines of pearl millet
Source: Breed Sci. 2024 Jul 5;74(3):240–6. doi: 10.1270/jsbbs.23082 (PMC11561417; doi:10.1270/jsbbs.23082)
Supplement: Supplementary file 1 — Supplemental Figures [file 74_240-s1.pdf]

doi: 10.1270/jsbbs.23082

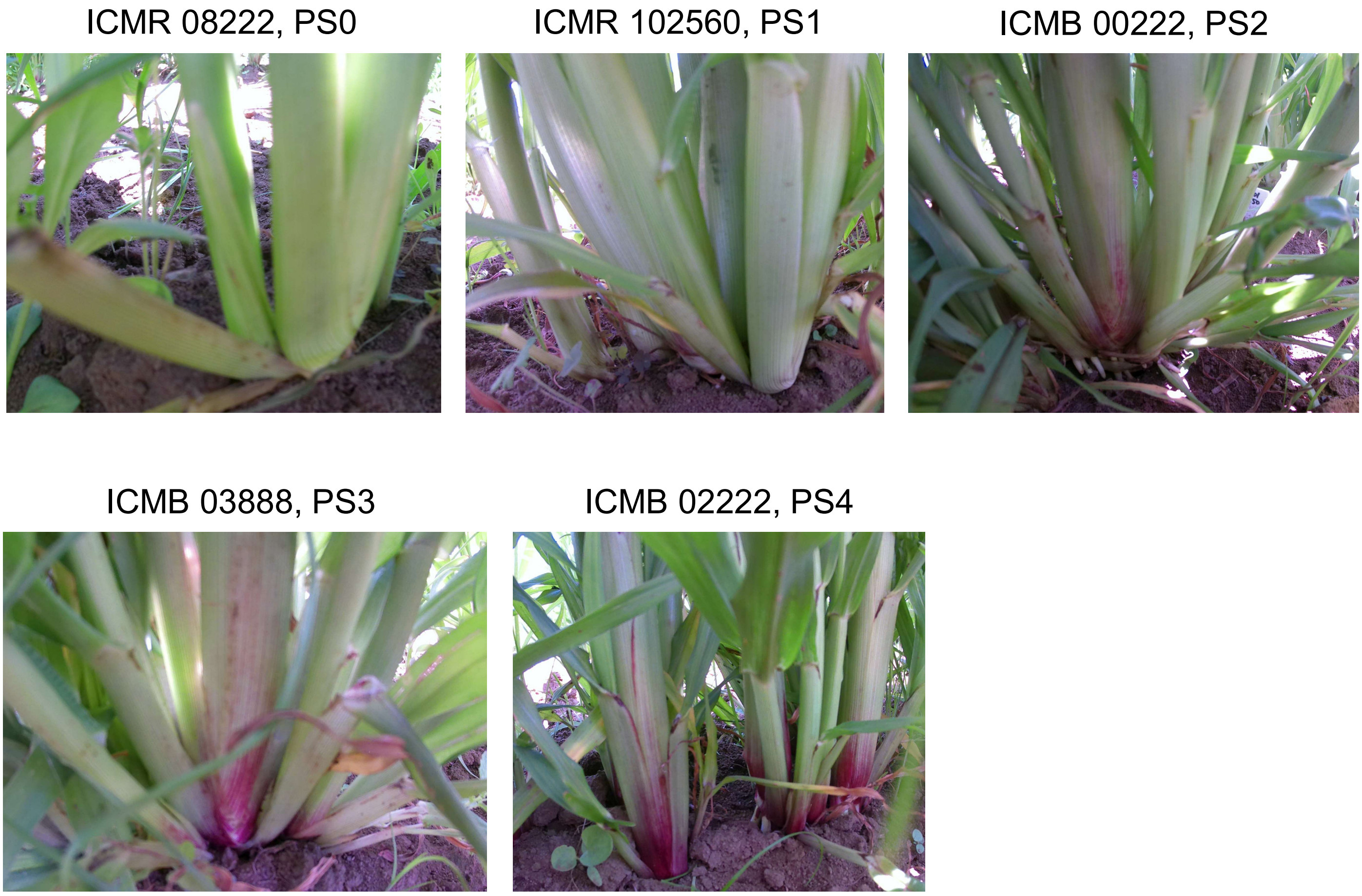

Supplemental Fig. 1. Examples of values of the shoot basal part (PS). On the top of each image, a line name and a corresponding PS value are presented. A representative image is presented for each line.

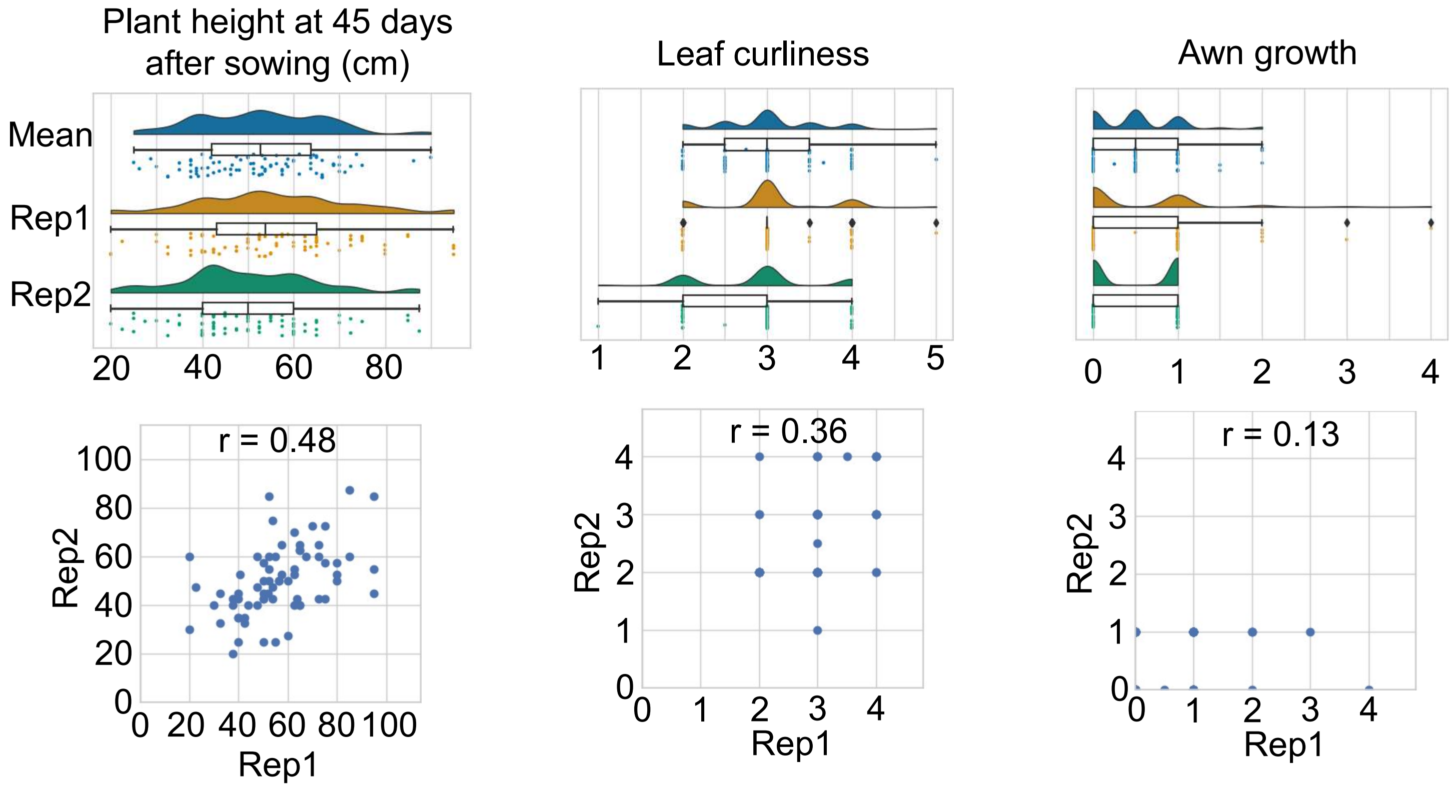

Supplemental Fig. 2. The distribution of phenotypic values and correlation between the two replicates for plant height at 45 days after sowing, leaf curliness, and awn growth.

Rep1, replicate 1; Rep2, replicate 2; Mean, the mean value of the two replicates.

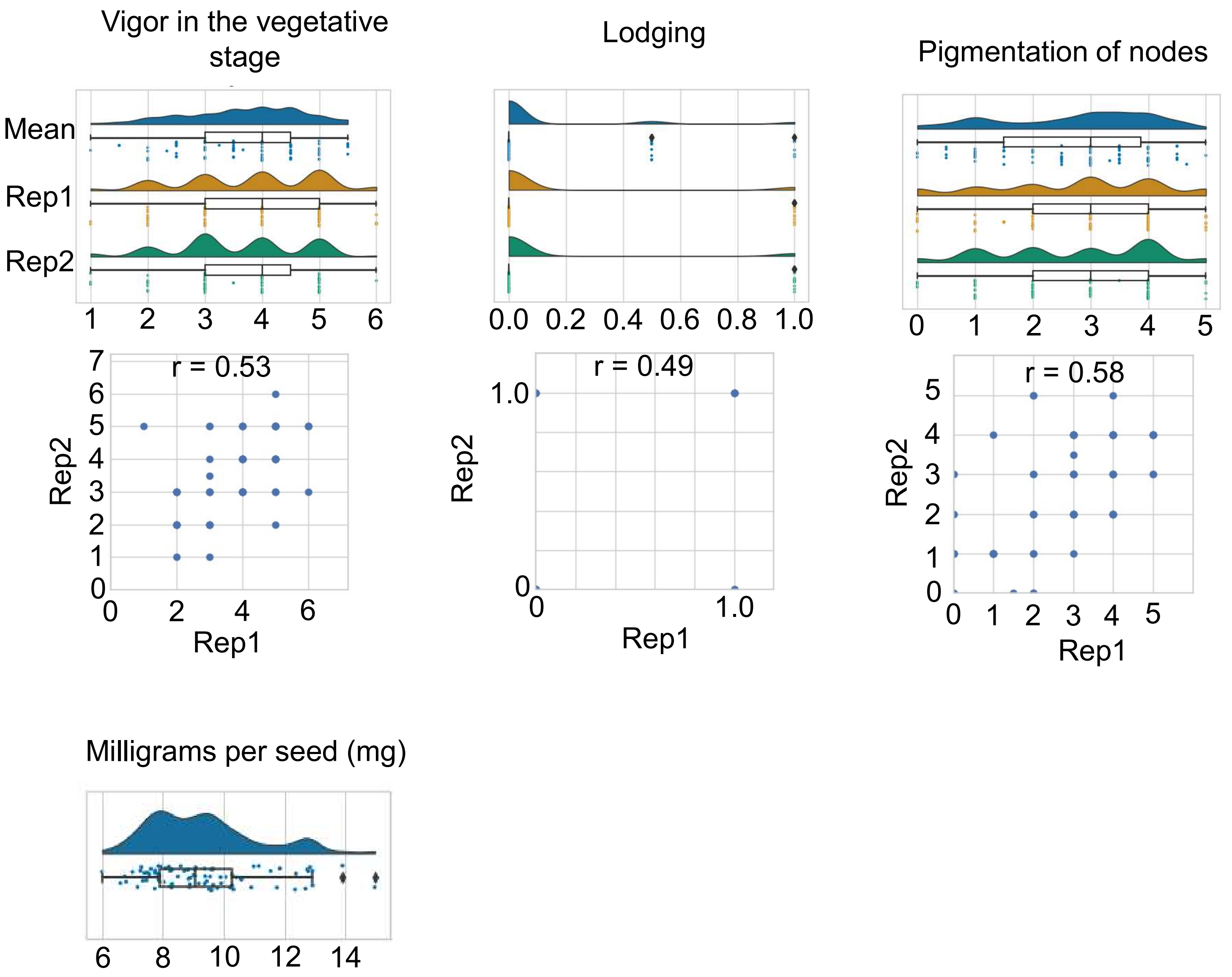

Supplemental Fig. 3. The distribution of phenotypic values and correlation between the two replicates for vigor in the vegetative stage, lodging, pigmentation of nodes, and milligrams per seed.

Rep1, replicate 1; Rep2, replicate 2; Mean, the mean value of the two replicates.

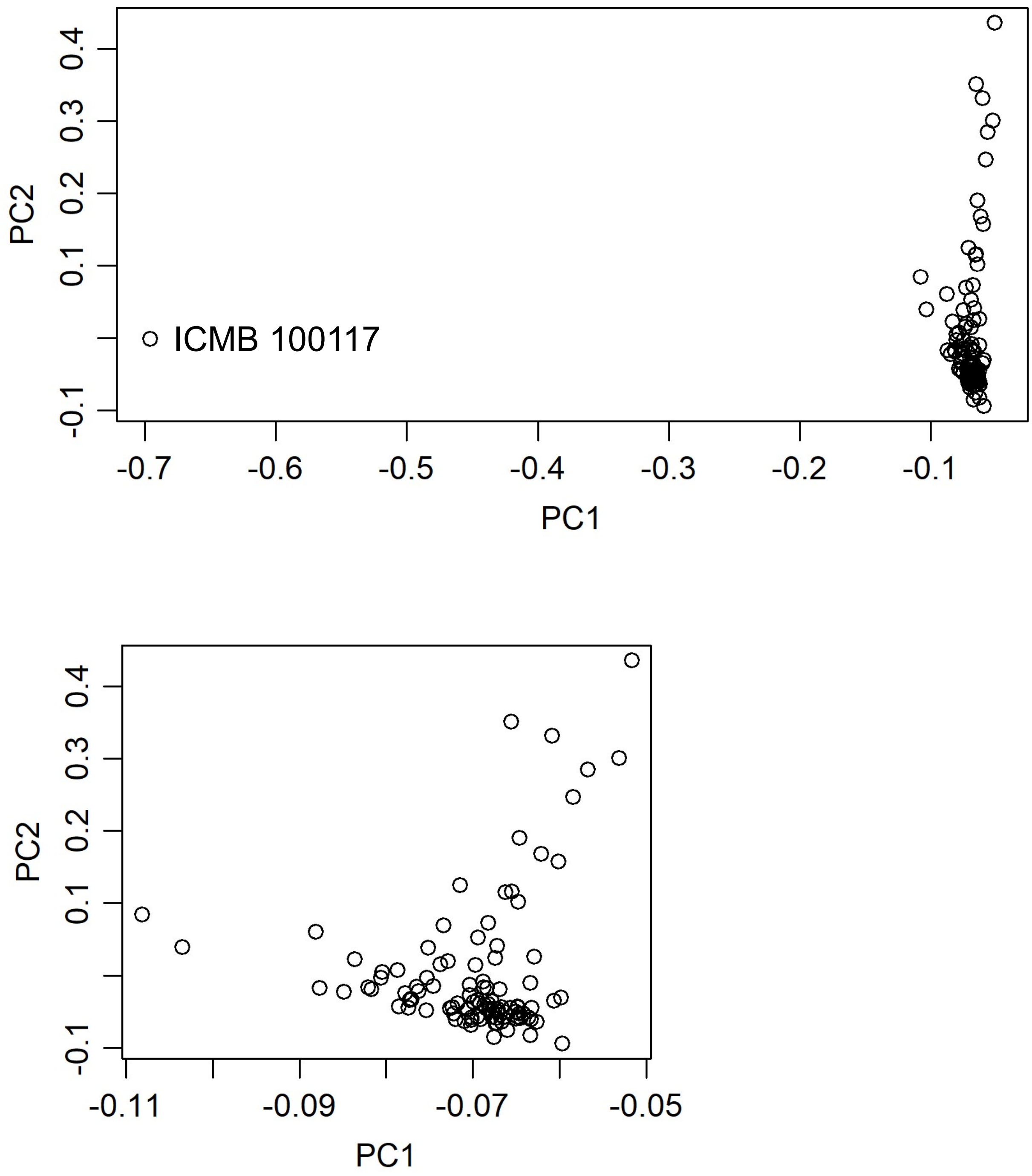

Supplemental Fig. 4. The plot between PC1 and PC2 obtained in the PCA using the genotypic data including the ICMB 100117 data (top panel) or excluding it (bottom).

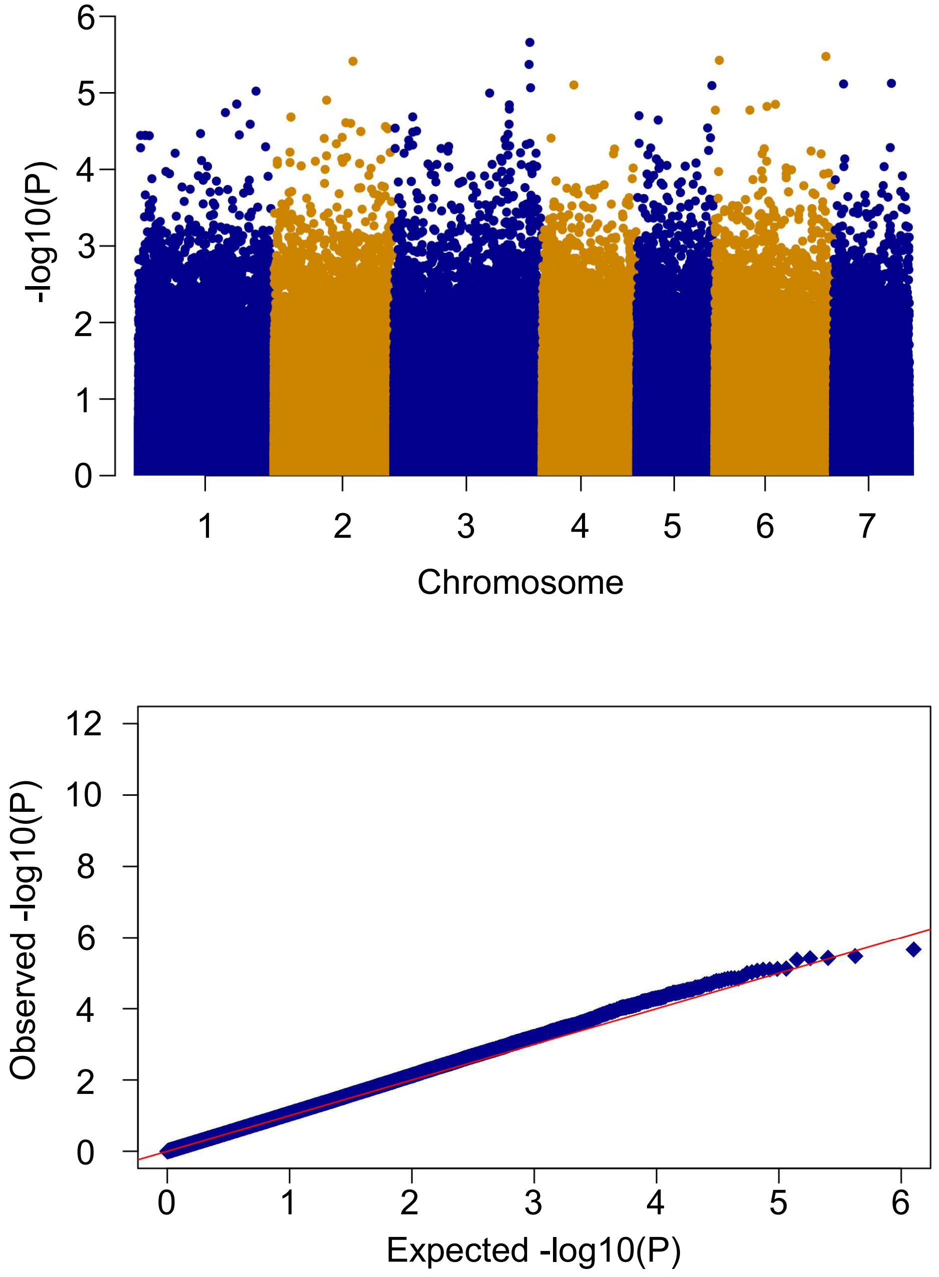

Supplemental Fig. 5. A Manhattan plot (top panel) and QQ plot (bottom) for the pigmentation of nodes (PN).
